# The learning curve of robotic cardiac surgery: a scoping review

**DOI:** 10.1007/s11701-025-02427-w

**Published:** 2025-08-11

**Authors:** Christina S. Boutros, Minji Jinny Kim, Mohammed El Diasty

**Affiliations:** 1 Department of Surgery, University Hospitals Cleveland Medical Center, Cleveland, OH USA; 2 Department of Medicine, University of Toronto, Toronto, Canada; 3 Department of Cardiac Surgery, University Hospitals Cleveland Medical Center, Cleveland, OH USA

**Keywords:** Robotic Surgery, Learning Curve, Outcomes, Cardiac Surgery

## Abstract

This systematic review aims to investigate the learning curve associated with robotic cardiac surgical procedures and its impact on operative efficiency and patient outcomes. An electronic search of MEDLINE, MEDLINE In‐Process, Embase, and the Cochrane Library databases was conducted in October 2023. Studies reporting outcomes of robotic cardiac surgical procedures during the early phase of the learning curve process were included. Intraoperative metrics and clinical outcomes were examined. Following the removal of duplicates, 2305 citations were screened, with 32 studies meeting inclusion criteria for full-text screening. Seven studies focused on totally endoscopic coronary artery bypass (TECAB), 12 on robotic mitral valve repair (MVR), and 8 on robotic coronary artery bypass grafting (CABG). Analysis revealed improved procedural efficiency along the learning curve, evidenced by reductions in surgical durations and operative complications. Notable enhancements were observed in total procedure time, bypass time, harvest time, and cross-clamp/occlusion time. Low mortality rates were consistently reported at both 30 days and 1-year post-surgery. As surgeons progress along the learning curve, there is a notable improvement in procedural efficiency and a reduction in adverse events. However, variability in the number of procedures required to attain proficiency suggests the influence of program size and individual surgeon experience. Standardized training protocols and ongoing mentorship are essential to optimize the learning curve while ensuring patient safety. Further research employing standardized metrics to define competency thresholds and expedite the learning process is warranted to enhance the proficiency of robotic cardiac surgeons.

**Supplementary Information:**

The online version contains supplementary material available at 10.1007/s11701-025-02427-w.

## Introduction

The utilization of robotics in surgery arose from the idea that while skilled surgeons may outperform robots in open surgeries, the constraints posed by minimally invasive surgery (MIS) require articulation and ergonomics not possible with the constraints of human anatomy [1]. Robotic cardiac surgery encompasses “any heart operation conducted with the assistance of robotic technology, whether entirely or partially” [2]. The advancement of robotic cardiac surgery is closely entwined with the progress in minimally invasive cardiac surgery (MICS) [1]. Early trailblazers led the way in pioneering off-pump coronary procedures through mini-thoracotomies, demonstrating decreased complications and increased cost-effectiveness [1, 3–6]. Shortly after, video-assisted mitral valve repair and replacement procedures using mini-thoracotomies and cutting-edge visualization technologies were developed [7, 8]. Despite initial skepticism and hurdles, the safety and effectiveness of MICS were affirmed through successful clinical series and randomized trials [9, 10]. Eventually, MICS was established as standard of care, laying the groundwork for the emergence and acceptance of robot-assisted cardiac surgery [1]. 

Robotic cardiac surgery offers patients numerous benefits, particularly in mitral valve and coronary revascularization procedures. These minimally invasive techniques, facilitated by robotics, result in smaller incisions compared to traditional sternotomy approaches, leading to reduced postoperative pain, shorter hospital stays, improved patient satisfaction, and faster recovery times [2, 11, 12]. Moreover, advancements in robotic technology allow for enhanced precision and visualization, enabling surgeons to operate on varied mitral pathologies more efficiently [2]. Despite initial concerns regarding longer procedure times and steeper learning curves, the evolution of robotic techniques has addressed these challenges, promising safer and more effective cardiac interventions for patients. Overall, the integration of robotics in cardiac surgery not only ensures better perioperative outcomes but also holds potential for further advancements in patient care through continued innovation and refinement of robotic-assisted procedures [13–15]. However, due to the learning curve associated with robotic cardiac surgery procedures, the true benefits of robotic techniques may not be readily apparent for surgeons that are amidst the learning process.

Understanding and navigating the learning curve in robotic cardiac surgery is crucial for accurately assessing its efficacy and ensuring optimal patient outcomes. As surgeons gain proficiency and experience, they can contribute to ongoing advancements in the field, ultimately enhancing the quality of care delivered to patients undergoing robotic-assisted cardiac procedures. Various metrics can be utilized to define learning curve, including the number of cases needed to attain optimal proficiency, alterations in operative variables over time, evaluating the time taken to reach a comparable level of competence to an established benchmark, or cumulative sum analysis (CUSUM) [16, 17]. While research has delved into the learning curve of robotic surgeries in gynecologic and urologic procedures, the delineation of the learning curve for robotic cardiac surgery remains inconclusive within the existing literature [18, 19].

Through an examination of the learning curve observed among cardiac surgeons integrating robotic methodologies into their clinical repertoire, this study endeavors to yield a comprehensive understanding of the learning dynamics’ impact on both surgical and patient-centric outcomes. Subsequently, this exploration aims to propose methodological refinements to optimize the learning curve, ensuring progressive advancement while safeguarding surgical efficacy and patient welfare.

## Methods

### Search strategy and study selection

This scoping review adhered to the Preferred Reporting Items for Systematic Reviews and Meta-Analysis (PRISMA) guidelines and was registered with OSF (https://osf.io/z4kax/?view_only=) [20]. Primary studies assessing the learning curve associated with various robotic cardiac surgeries were systematically searched in various electronic databases including MEDLINE, MEDLINE In‐Process, Embase, and the Cochrane Library (up to March 14th, 2024). Google scholar was manually searched to complement the electronic search. A combination of keywords such as “Robotic”, “Cardiac”, “Surgery”, and “Learning Curve” guided the literature search. Eligible articles were required to be written in English, constitute primary studies involving adults aged 18 years and above, and report information regarding metrics associated with the learning curve for robotic cardiac surgery. Case reports, opinion articles, or review articles were excluded. Studies evaluating transcatheter aortic valve implantation, MitraClips, and endoscopic surgical procedures were also excluded.

*Study Outcomes:* The primary outcome of interest included case number to overcome the early learning curve. Secondary outcomes included morbidity, mortality, and post-surgery outcomes such as stroke, heart failure, renal failure, procedure length, and length of stay (LOS).

*Screening:* Literature search results were uploaded to Covidence review software (Covidence Systematic Review Software, Veritas Health Innovation, Melbourne, Australia. http://www.covidence.org).

*Data Extraction*: Author, country of origin, publication year, study design, patient demographics, patient age, study methodology, number of robotic surgeons involved, surgeon’s experience level, procedures performed, robotic technology utilized, number of procedures required to overcome the learning curve, metrics used to assess the learning curve, as well as clinical outcomes data were amongst the data extracted. Two reviewers independently, and in duplicate, assessed all of the studies’ quality using the Newcastle Ottawa Quality Assessment. Any conflicts or discrepancies were settled by a third senior reviewer.

## Results

**Fig. 1:**
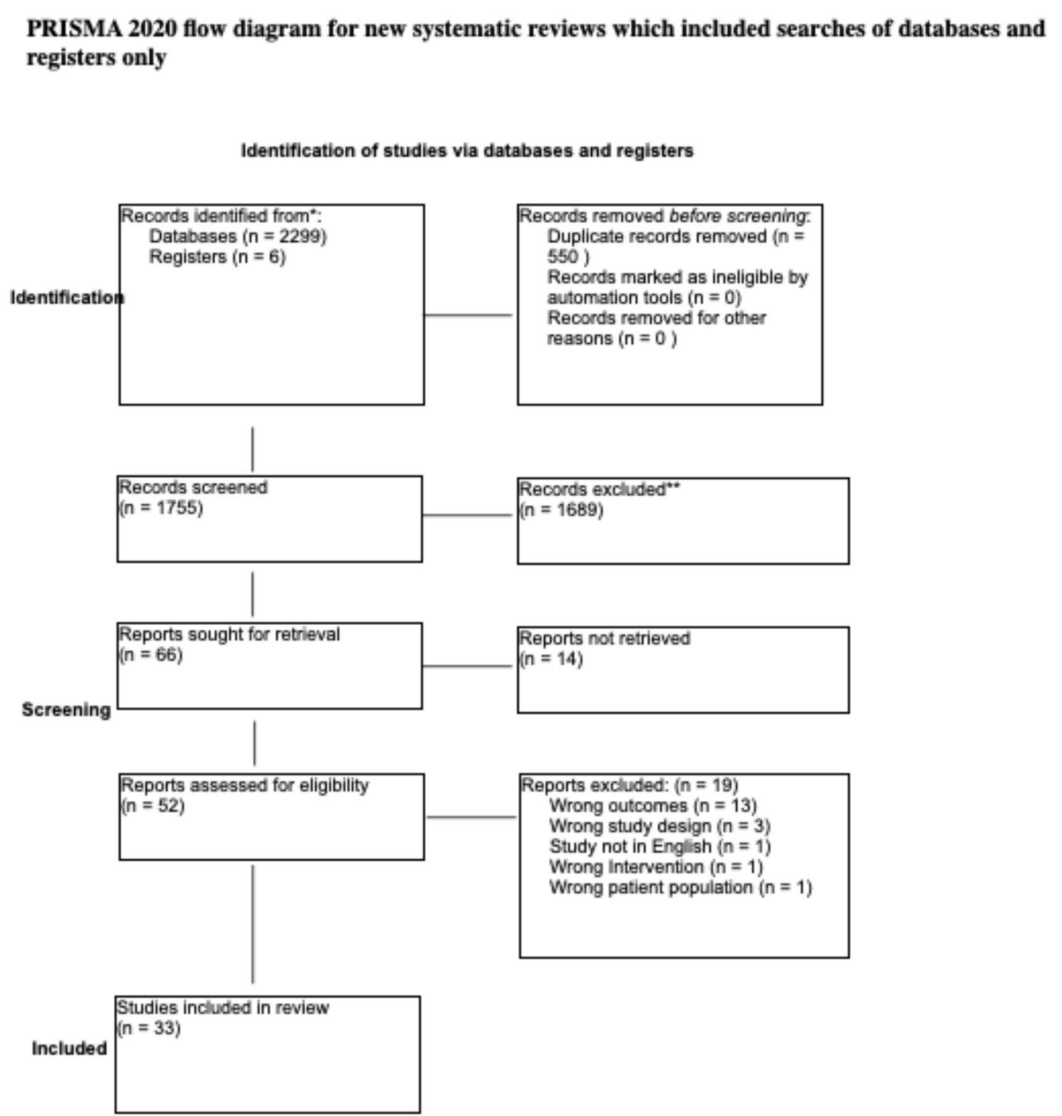
PRISMA Diagram

The search generated 2305 results, of which 550 were removed as duplicates. Of the 1756 screened articles, 1689 were excluded from title/abstract screening. The remaining 52 articles were retrieved for full-text review, 20 of which were excluded because they did not evaluate the predetermined outcome measures (n = 13), did not evaluate robotic surgical techniques (n = 4), did not evaluate adult patients undergoing robotic cardiac surgery (n =1), were an opinion article, review article, or case report (n = 3), or was not written in English (n =1). Data was captured for a total of 32 studies in a prespecified grid. A PRISMA diagram can be found in Fig. 1.

### General characteristics

The included studies were published between 2001 and 2023. Data was analyzed prospectively in 19 studies (59%) [21–38] and retrospectively in 13 studies (41%) [39–51]. Majority of the studies (n=30) were single arm studies, commonly comparing between quartiles or tertiles, while only two studies compared to control or conventional surgery. Among the 32 eligible studies, 18 studies reported the number of robotic surgeons; two studies included more than ten robotic surgeons [31, 34]. Moreover, 14 studies detailed the years of surgical experience of participating surgeons, and from these studies, there was wide variability in each surgeons’ level of experience, ranging from no initial experience [34, 48, 51], to others who had over 650 cases of experience prior to the start of the study [25] most of whom were performing mitral valve repairs (n=14) or CABG procedures (n=13).The Da Vinci surgical system (Si or Xi) was used in 27 studies (85%), while 2 studies used the Zeus surgical systems (6%). The remaining 3 studies (15%) did not report what robotic surgical system was used (Table 1).

**Table 1:** General characteristics of included studies (N=32)

Study	Country	Year(s)	Study design	Study arms	Number of robotic surgeons	Surgeons’ level of experience with robotic surgery	Procedures/ tasks performed	Robotic technology used	Learning curve overcome	Number of cases to overcome learning curve
Argenziano 2006	USA	2006	Retrospective	Single	NR	5 predefined stages of TECAB training requirements	Totally endoscopic coronary artery bypass (TECAB)	da Vinci	No	NR
Barac 2021	USA	2021	Retrospective	Single	6	Academic medical center with vast experience in endoscopic, non-robotic, and robotic mitral repairs	Mitral valve repair (MVR)	da Vinci	No	16
Bonaros 2006	Austria	2006	Prospective	Single	NR	NR	Atrial septal defect (ASD) repair	da Vinci	No	NR
Bonatti 2009	Austria	2009	Prospective	Single	1	NR	TECAB	da Vinci	Yes	>100
Bonatti 2004	Austria	2004	Prospective	Single	NR	NR	TECAB	da Vinci	No	NR
Bonatti 2008	Austria	2008	Prospective	Single	NR	NR	TECAB	da Vinci	NR	25
Charland 2011	USA	2011	Retrospective	Single	NR	NR	MVR	da Vinci	NR	NR
Cheng 2014	China	2014	Prospective	Single	1	Prior experience with successfully performing >650 cases of robotic cardiac surgery at a single center	TECAB	da Vinci	No	NR
ChitwoodJr 2005	USA	2005	Retrospective	Single	1	NR	MVR	da Vinci	No	NR
ChitwoodJr 2001	USA	2001	Prospective	Single	NR	NR	MVR	da Vinci	No	NR
Gao 2012	China	2012	Prospective	Single	NR	NR	MVR	da Vinci	No	NR
Goodman 2017	USA	2017	Prospective	Single	2	Surgeon A performed approx. 25 robotically assisted cases CABG. Surgeon B had no previous robotic experience	MVR	NR	No	NR
Gullu 2021	Turkey	2021	Retrospective	Single	NR	NR	MVR	da Vinci	Yes	30
Hemli 2013	USA	2013	Prospective	Single	NR	NR	Minimally invasive CABG	da Vinci	Yes	20
Isgro 2003	Germany	2003	Prospective	Single	NR	NR	Internal mammary artery takedown	Zeus	No	NR
Jones 2005	USA	2005	Retrospective	Single	1	Surgeon had performed well over 40 robotically-assisted internal thoracic artery harvests	MVR	da Vinci	No	NR
Kakuta 2020	Japan	2020	Retrospective	Single	3	NR	MVR	da Vinci	Yes	10
Kesavuori 2018	Finland	2018	Retrospective	Comparative (control)	NR	NR	MVR	da Vinci	Yes	30
Klepper 2022	Belgium	2022	Retrospective	Single	4	NR	MVR	da Vinci	NR	NR
Masroor 2021	USA	2021	Prospective	Single	114	No prior experience	Coronary artery bypass graft (CABG)	NR	Yes	8–10
Novick 2003	Canada	2003	Prospective	Single	NR	NR	CABG	Zeus	No	18–20
Oehlinger 2007	Austria	2001- 2005	Prospective	Single	NR	NR	CABG	da Vinci	Yes	50
Patrick 2021	USA	2014- 2019	Prospective	Single	114	No prior experience	CABG	NR	Yes	10
Ramzy 2014	USA	2005- 2012	Retrospective	Single	2	No prior experience	MVR	da Vinci	Yes	120
Sagbas 2006	Turkey	2006	Prospective	Single	NR	NR	CABG, ASD closure	da Vinci	Yes	59
Schachner 2011	Austria	2001- 2009	Retrospective	Single	3	No prior experience	TECAB	da Vinci	Yes	20
Schachner 2009	Austria	2001- 2008	Retrospective	Single	2	Completed TECAB training before robotic surgeries	TECAB, MIDCAB, CABG	da Vinci	No	NR
Seo 2019	USA	2008- 2016	Prospective	Comparative (conventional surgery)	1	Prior experience with 100 robotically-assisted cases	MVR	da Vinci	Yes	100
VandenEynde 2021	Belgium, USA	2015- 2020	Retrospective	Single	3	NR	Single internal mammary artery bypass grafting	da Vinci	Yes	100
Xiao 2014	China	2007- 2013	Prospective	Single	1	No prior experience	ASD repair	da Vinci	Yes	60–120
Yaffee 2014	USA, Czech Republic	NR	Prospective	Single	2	Completed clinical scenarios, simulations, wet laboratories, and ‘‘expert’’ observation for 3 months	MVR	da Vinci	Yes	NR
Jonsson 2023	USA	2009- 2020	Prospective	Single	1	Completed preliminary training.	CABG	da Vinci	Yes	250-500

*NR* not recorded. Assumption that a plateau of the learning curve means the curve has been overcome, as no further improvement is being made

### Learning curve metrics

**Fig. 2:**
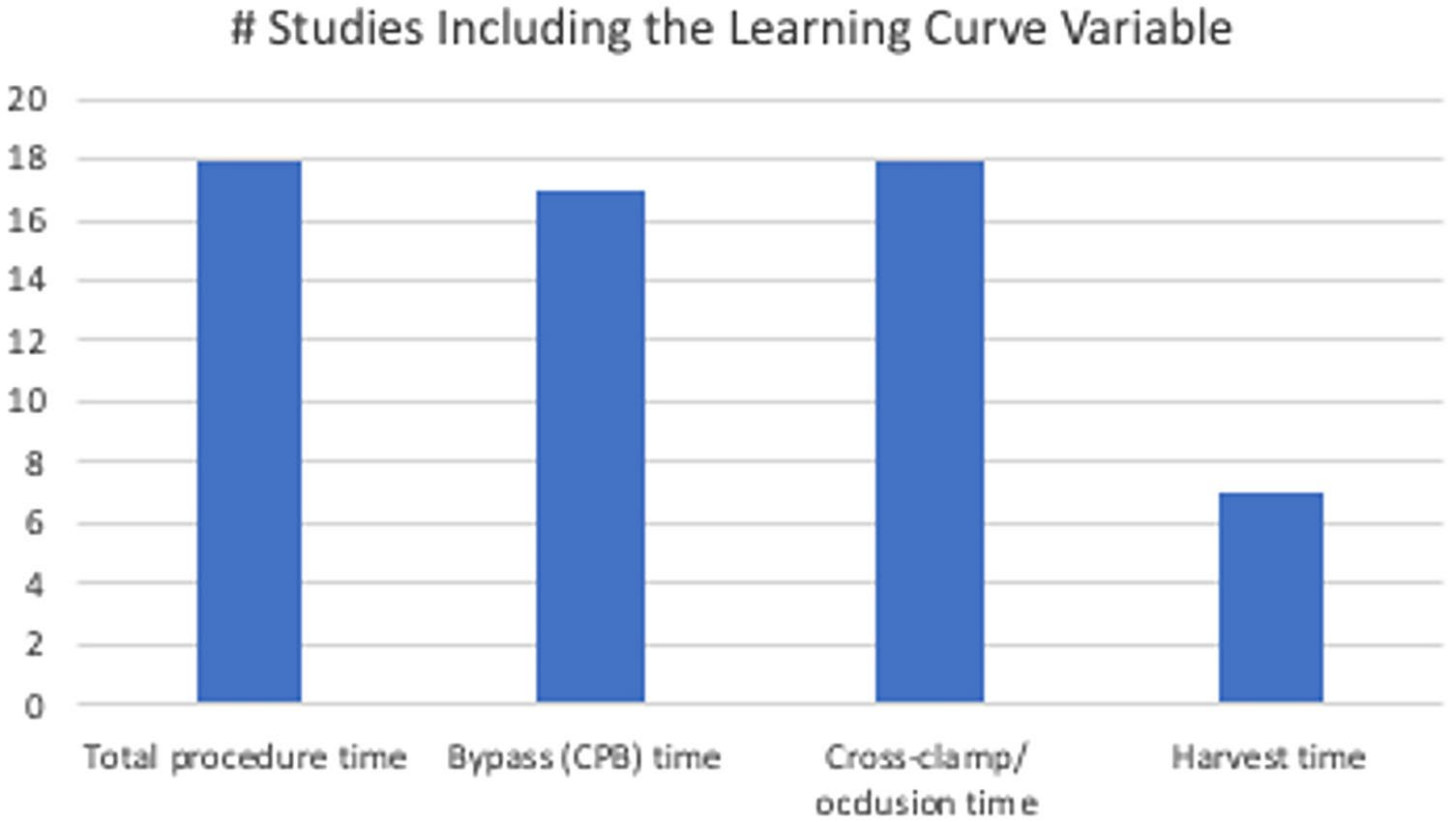
Frequency of Time-Based Metrics Used to Define the Learning Curve in Included Studies. This figure summarizes the number of studies that reported key intraoperative time metrics to assess learning curve progression. Total procedure time and cross-clamp/occlusion time were each reported in 18 studies, cardiopulmonary bypass (CPB) time in 17 studies, and harvest time in 7 studies. These variables represent the most commonly used quantitative indicators of surgical proficiency in robotic cardiac surgery learning curve analyses

Time-based metrics were the most frequently reported variables used to evaluate the learning curve in robotic cardiac surgery. The most commonly reported time-based metrics used to assess the learning curve across studies were total procedure time, cross-clamp/occlusion time, and cardiopulmonary bypass (CPB) time, followed by harvest time. These variables were used frequently as surrogate indicators of operative efficiency and technical proficiency (Fig. 2). Of the 32 studies included in the analysis, 26 (81%) reported at least one time-based metric at two different time points, with operative time being the most commonly reported time-based metric (n=16), followed by time on cardiopulmonary bypass (CPB) (n=15). Three studies utilized cumulative sum control chart analysis (CUSUM) to analyze the learning curve, which is a method that helps detect changes in a process by monitoring the cumulative sum of deviations from a reference value, making it a valuable tool for quality control and process improvement [32, 38, 50]. The learning curve was overcome in 16 studies (50%); the number of procedures required to overcome the learning curve was defined in 18 studies (56%), ranging widely from 10 to over 250 procedures (Table 1).

### Procedural time metrics

Various time metrics were used to evaluate the learning curve, including change in total operative time (n=16), CPB time (n=15), cross-clamp time (n=13), and artery/vein harvest time (n=7). As surgeons progressed across the learning curve, overall, there was a mean reduction across the learning curve (84.7 minutes, 32.6 minutes, 18.1 minutes, 41.6 minutes, respectively; Table 2). When comparing metrics before and after achieving the learning curve, the mean total operative time was 304.7 minutes and 220.0 minutes, respectively. The mean CPB time before overcoming the learning curve was 157.9 minutes, which decreased to 125.3 minutes after overcoming the learning curve. There was also a recorded reduction in mean cross-clamp/occlusion time after overcoming the learning curve (108.5 min vs. and 90.4 min). Additionally, the mean harvest time decreased from 82.7 minutes to 41.1 minutes. The reported values can be found in Table 2, 3.

**Table 2:** Time-based metrics to evaluate learning curve (N=26)

Study	Operative time before	Operative time after	CPB time before	CPB time after	Cross-clamp/ occlusion time before	Cross-clamp/occlusion time after	Harvest time before	Harvest time after
Argenziano 2006	400.0	260.0	130.0	117.0	70.0	71.0	62.0	60.0
Barac 2021	NR	NR	265.0	265.0	146.0	146.0	NR	NR
Bonaros 2006	400.0	314.0	220.0	144.0	115.0	69.0	NR	NR
Bonatti 2009	400.0	272.0	140.0	89.0	81.0	47.0	NR	NR
Bonatti 2004	180.0	50.0	NR	NR	NR	NR	180.0	50.0
Bonatti 2008	540.0	360.0	NR	NR	NR	NR	NR	NR
Cheng 2014	179.0	157.6	NR	NR	NR	NR	29.6	25.3
ChitwoodJr 2005	84.0	66.0	174.0	150.0	138.0	114.0	NR	NR
ChitwoodJr 2001	114.0	108.0	204.0	174.0	156.0	144.0	NR	NR
Goodman 2017	414.0	364.0	148.0	102.0	NR	NR	NR	NR
Gullu 2021	NR	NR	155.3	118.9	102.3	80.0	NR	NR
Hemli 2013	414.0	41.6	NR	NR	NR	NR	39.0	30.3
Isgro 2003	NR	NR	NR	NR	NR	NR	95.0	44.0
Jones 2005	NR	NR	152.0	123.0	119.0	89.0	NR	NR
Kesavuori 2018	277.0	250	171 .0	151.0	111.0	101.0	NR	NR
Klepper 2022	309.2	269.8	162.1	155.5	116.7	108.4	NR	NR
Masroor 2021	NR	NR	91.8	84.1	NR	NR	NR	NR
Novick 2003	537 .0	472.0	NR	NR	NR	NR	NR	NR
Oehlinger 2007	NR	NR	NR	NR	NR	NR	48.0	42.0
Patrick 2021	NR	NR	91.8	84.1	NR	NR	NR	NR
Ramzy 2014	NR	NR	NR	NR	116.0	91.0	NR	NR
Sagbas 2006	NR	NR	NR	NR	NR	NR	125.0	20.0
Schachner 2009	NR	NR	115.0	105.0	67.0	70.0	NR	NR
VandenEynde 2021	249.0	259.0	NR	NR	NR	NR	NR	NR
Xiao 2014	420.0	150.0	95.0	47.0	72.0	35.0	NR	NR
Jonsson 2023	195.0	176.0	NR	NR	NR	NR	NR	NR

Charland 2011, Gao 2012, Kakuta 2020, Schachner 2011, Seo 2019, Yaffee 2014 excluded

**Table 3:** Pooled Mean Procedural Times Before and After Learning Curve Completion

	Before learning curve	After learning curve	Difference/reduction
Operative time	304.7	220	84.7
CPB time	157.9	125.3	32.6
CC time	108.5	90.4	18.1
Harvest time	82.7	41.1	41.6

Means, minutes

This table summarizes the average operative, cardiopulmonary bypass (CPB), cross-clamp (CC), and graft harvest times across studies that reported time-based metrics before and after the defined learning curve threshold. Values reflect pooled means in minutes and illustrate improvements in procedural efficiency associated with learning curve progression

### Post-Surgical quality metrics

To further characterize the clinical impact of the learning curve, we also examined secondary outcome measures including conversion to open access, reoperation for bleeding, perioperative mortality, ICU length of stay, and total hospital length of stay. Many studies demonstrated favorable trends in these metrics as surgical teams progressed beyond the learning curve threshold, suggesting that improvements in technical performance may also enhance patient safety and recovery (Table 4). As surgeons progress through the learning curve there is a statistically significant decrease in ICU LOS, ranging from 1 to 8 hours. Moreover, the mean hospital LOS was reported in 5 studies (10.70 days [SD 4.53 days]), which demonstrated a decrease to a mean of 8.54 days after the learning curve was overcome.The mortality rate, both 30 day and 1-year mortality, were low amongst all studies in this review. Other reported complications included renal failure, stroke, bleeding, infection, and arrhythmia. The frequency of occurrence of each complication varied across the studies (Table 4).

**Table 4:** Additional metrics used to evaluate learning curve (N=31)

Study	Conversion to open access	Reopening for bleeding	Mortality	ICU LOS (hours)	Total hospital LOS (days)
Argenziano 2006	5/85 (6%)	0	0	35.0	5.1
Barac 2021	1/133 (1%)	0	0	NR	5
Bonaros 2006	0	0	0	26.0	8
Bonatti 2009	7/25 (28%) in phase 1, 2/ 25 (8%) in phase 2, 1/25 (4%) in phase 3, and 1/25 (4%) in phase 4	4/ 25 (16%) in phase 1, 3/25 (12%) in phase 2, 1/25 (4%) in phase 3, and 0/ 25 (0%) in phase 4	0	Phase 1 = 23, phase 2 = 20, phase 3 =20, phase 4 = 19	Phase 1 = 7, phase 2 = 6, phase 3 =5, phase 4 = 6
Bonatti 2004	2	3	0	24.0	8
Bonatti 2008	9%	5%	0	19	6
Cheng 2014	0	0	0	Quintile 1 = 27.2 ± 20, quintile 2 = 25.4 ± 21 quintile 3 = 26.4 ± 20	NR
ChitwoodJr 2005	0	2	1	NR	4.8
ChitwoodJr 2001	NR	NR	NR	19.3	3.5
Gao 2012	0	0	0	1.5	NR
Goodman 2017	NR	15 (3.7%)	0	32 to 28 to 24 hours (13% and 25% decrease)	5.2 to 4.5 to 3.8 days (13% and 27% decrease)
Gullu 2021	0	3.3%	0	23.2 to 19.5	NR
Hemli 2013	0	NR	0	NR	NR
Isgro 2003	NR	0	NR	NR	NR
Jones 2005	3	0	2 (6%)	NR	first 12: 5.7 last 12: 4.8 last 5: 3.4
Kakuta 2020	1	0	0	48	7
Kesavuori 2018	14 (9.9%)	2 (1.4%)	1 (0.7%)	24	7
Klepper 2022	5 (2.2%)	NR	0	57.6	7.9
Masroor 2021	54 (4.5%)	NR	10 (0.8%)	NR	2 (0.2%)
Novick 2003	16 (17.8%)	2	0	28.8	4
Oehlinger 2007	1	0	0	20	7
Patrick 2021	Group 1 (n=465): 36 (7.7) Group 2 (n=730): 18 (2.5)	Group 1 (n=465): 88 (18.9%) Group 2 (n=730): 79 (10.8%)	Group 1 (n=465): 5 Group 2 (n=730): 5	NR	NR
Ramzy 2014	1	7	0	NR	5.8
Sagbas 2006	2	2	NR	29.3	8.1
Schachner 2011	46	NR	2 (0.6%)	20	6
Schachner 2009	1	NR	0	NR	20
Seo 2019 (open vs robotic)	NR	4 vs. 3	7 vs. 1	144 vs. 84	9.9 vs. 6.5
VandenEynde 2021	NR	NR	6	NR	NR
Xiao 2014	0	0	0	29	12
Yaffee 2014	0	0	NR	NR	NR
Jonsson 2023	1.6%	22 (2.2%)	6 (0.6%)	37.7	4.43

Charland 2011 excluded

## Discussion

Our systematic review highlights the complexities associated with the learning curve (LC) in robotic cardiac surgery and its broader implications for patient safety, surgeon proficiency, and healthcare administration. Robotic surgery offers significant advantages in terms of precision and reduced invasiveness, but mastering these techniques requires considerable time and resources. The process of understanding and overcoming the LC is critical not only for safeguarding patient outcomes but also for optimizing surgical techniques and program implementation. 

Assessing the LC involves delineating various metrics that reflect skill acquisition and procedural proficiency. Parameters such as operative time, complication rates, and outcomes serve as crucial indicators in evaluating the progression along the LC trajectory. However, the challenge lies in standardizing these metrics across different studies to facilitate meaningful comparisons and draw robust conclusions. Among the included studies, the definition of the learning curve and criteria for overcoming it varied considerably. Some studies used procedural time metrics—such as reductions in total operative time, cardiopulmonary bypass time, cross-clamp time, or graft harvest time—as indicators of proficiency. Others applied cumulative sum (CUSUM) analysis or relied on case count thresholds, complication rates, or conversion to open surgery to delineate when the learning curve had been surpassed. A small number of studies used qualitative criteria, including surgeon-reported comfort, technical ease, or procedural consistency. This heterogeneity is reflected in the reported number of cases required to overcome the learning curve, which ranged widely from fewer than 10 cases to over 500. This variation likely reflects differences in procedural complexity, surgeon baseline experience, institutional volume, and access to structured training resources such as simulation or mentorship programs.

Overcoming the LC entails multifaceted approaches, including structured training programs in specialized centers, mentorship by experienced robotic surgeons, and meticulous case selection. [52] Emerging technologies, such as high-fidelity robotic simulators and AI-guided performance feedback systems, may further enhance skill acquisition during early training and shorten the learning curve. Leveraging resources like the AATS Foundation robotics fellowship can further enhance the learning experience and accelerate the LC progression, as it aims to standardize skill acquisition and reduce variability in early training experiences. Studies have suggested that high-volume centers may provide more consistent exposure to robotic procedures and refined intraoperative workflows, potentially accelerating the learning process compared to low-volume institutions [23].

The learning process for robotic cardiac surgery is not limited to surgeons but extends to the entire surgical team, including anesthesiologists, perfusionists, and nurses. As robotic programs are adopted, hospitals must provide comprehensive team training to ensure optimal outcomes. Insights from our analysis suggest that structured training programs, mentorship from experienced robotic surgeons, and careful case selection are critical for successfully navigating the LC.

The reported discrepancies in the number of cases required to overcome the LC underscore the multifactorial nature of skill acquisition in robotic surgery. Factors such as individual surgeon experience, procedural volume, and institutional resources, such as wet lab, simulation, proctors, and mentors all play pivotal roles in influencing the trajectory of the LC [53, 54]. Our analysis of the time metrics used to assess learning curves revealed significant reductions in total procedure time, bypass time, harvest time, and cross-clamp/occlusion time after the learning curve was achieved. This is consistent with current literature which suggests that less time is required to perform robotic surgeries as surgeons gain more experience and thus improve their proficiency, underscoring the merit in providing surgeons with ample opportunities to perform procedures utilizing robotic technology [28, 55, 56].

Most importantly, our findings suggest that gaining proficiency improves patient outcomes, leading to shorter surgeries and reduced operative complications. Although the average hospital LOS varied between studies, there were significant reductions in hospital stay after surgeons achieved peak proficiency on the learning curve, which was noted. Moreover, close monitoring of patient safety across various stages of the learning curve should be prioritized, as the existing evidence, although limited, indicates a potential higher occurrence of adverse patient safety events during the early phases of robotic surgery [57, 58]. Due to the wide heterogeneity in surgical procedures, study designs, and learning curve definitions across the literature, establishing a universal cutoff for proficiency (e.g., a specific number of cases) is not currently feasible. However, recognizing the need for more consistency, we propose a conceptual framework based on the most commonly reported metrics — including reductions in operative time, complication rates, and application of cumulative sum (CUSUM) analysis — as a potential foundation for future consensus-building efforts. This framework aims to guide the development of standardized benchmarks in future studies while acknowledging current variability.

## Limitations

Our study has several limitations that warrant consideration. Firstly, most included studies were single-arm and single-center studies, which may limit the generalizability of the findings. Only English-language studies were included, as none of the authors are fluent in other languages. While this may introduce language bias, inclusion of studies that could not be critically appraised would compromise the methodological integrity of the review. There exists substantial heterogeneity in the methodologies employed across these studies for assessing the learning curve (LC), including variations in the definitions used for key terms such as “proficiency” and “satisfaction”, and the criteria delineating the point of overcoming the LC. This variability extends to outcome measures, impeding direct comparisons between studies. Furthermore, our study did not account for the baseline experience levels of individual surgeons, which could potentially confound the observed LC trajectories. Additionally, this review was limited to studies evaluating robotic cardiac surgery and did not include comparator arms involving conventional or open surgical approaches. This was a deliberate methodological choice to maintain a focused scope, though future comparative analyses may help contextualize the learning curve differences across surgical modalities.

## Conclusion

In conclusion, while robotic cardiac surgery continues to advance, the learning curve associated with its adoption remains highly variable and not yet clearly defined. Our review demonstrates that as surgeons overcome the learning curve, there are consistent improvements in procedural efficiency and patient outcomes. To support safe and effective adoption, we recommend the use of operative time and complication rates as practical surrogate indicators of proficiency. Structured training environments—including simulation platforms, formal mentorship, and dedicated fellowships—may help accelerate skill acquisition and ensure patient safety. Future research should aim to identify institutional factors that facilitate efficient learning, explore how different training models influence outcomes, and work toward the development of standardized learning curve benchmarks that can be applied across surgical programs.

## Supplementary Information

Below is the link to the electronic supplementary material.

Supplementary file1 (DOCX 18 KB)

## Data Availability

No datasets were generated or analysed during the current study.

## Abbreviations

MVR: Mitral Valve Repair
TECAB: Totally Endoscopic Coronary Artery Bypass Graft
CABG: Coronary Artery Bypass Graft
MICS: Minimally Invasive Cardiac Surgery
PRISMA: Preferred Reporting Items for Systematic Reviews and Meta-Analysis
CUSUM: Cumulative Sum Analysis
LOS: Length of Stay
CBP: Cardiopulmonary Bypass
ICU: Intensive Care Unit
CC: Cross-clamp
