# Supplementary material for: The learning curve of robotic cardiac surgery: a scoping review
Source: J Robot Surg. 2025 Aug 11;19(1):476. doi: 10.1007/s11701-025-02427-w (PMC12339607; doi:10.1007/s11701-025-02427-w)
Supplement: Supplementary file 1 — Supplementary file1 (DOCX 18 KB) [file 11701_2025_2427_MOESM1_ESM.docx]

Supplemental Table 1. Standardized Definitions of Learning Curve Terminology in Robotic Cardiac Surgery

| Term | **Definition** |
| --- | --- |
| Learning Curve (LC) | The process by which a surgeon or surgical team acquires proficiency in a procedure, typically measured over a series of consecutive cases. |
| Proficiency | A level of operative performance at which consistent outcomes and procedural efficiency are achieved. Often defined by stable or reduced operative time, low complication rates, or a plateau on a CUSUM plot. |
| Learning Curve Threshold | The number of cases required to reach proficiency. This threshold varies widely in the literature and may be determined by statistical methods (e.g., CUSUM), expert consensus, or empirical trends. |
| CUSUM Analysis | A statistical technique (Cumulative Sum Control Chart) used to detect shifts in performance by monitoring deviations from a reference value across a sequence of cases. Often used to define LC thresholds. |

Supplemental Table 2. Search strategy executed October 2, 2023

Database: MEDLINE, MEDLINE In‐Process, MEDLINE Epub Ahead of Print and Embase <1946 to October 1, 2022>

1. exp Robotic Surgical Procedures/ or robot surgical procedures.mp. (14109)
2. robotics.mp. or exp Robotics/ (44195)
3. ((surg* or procedur* or navigat*) adj3 (comp* or image*)).mp. (270919)
4. computer assisted surgery.mp. or exp Surgery, Computer-Assisted/ (36300)
5. 1 or 2 or 3 or 4 (309873)
6. thoracic surgery.mp. or exp Thoracic Surgery/ (32034)
7. coronary artery bypass.mp. or exp Coronary Artery Bypass/ (71100)
8. heart valve prosthesis.mp. or exp Heart Valve Prosthesis/ (56305)
9. ((cardiac or heart) adj2 surg*).mp. (103093)
10. 6 or 7 or 8 or 9 (231883)
11. learning curve.mp. or exp Learning Curve/ (14287)
12. train*.mp. (704443)
13. proficien*.mp. (27958)
14. competen*.mp. (261929)
15. skill*.mp. (266897)
16. 11 or 12 or 13 or 14 or 15 (1097005)
17. 5 and 10 and 16 (962)

Database: Embase <1974 to 2022 October 1>

1. robot assisted surgery.mp. or exp robot assisted surgery/ (23003)
2. robotics.mp. or exp robotics/ (49855)
3. ((surg* or procedur* or navigat*) adj3 (comp* or image*)).mp. (392610)
4. computer assisted surgery.mp. or exp computer assisted surgery/ (33383)
5. 1 or 2 or 3 or 4 (452640)
6. heart surgery.mp. or exp heart surgery/ (440799)
7. coronary artery bypass graft.mp. or exp coronary artery bypass graft/ (88676)
8. heart valve prosthesis.mp. or exp heart valve prosthesis/ (46107)
9. (cardiac adj2 surg*).mp. (87356)
10. 6 or 7 or 8 or 9 (461794)
11. learning curve.mp. or exp learning curve/ (25736)
12. train*mp. (1010347)
13. proficien*.mp. (36811)
14. competen*.mp. (285830)
15. skill*.mp. (347981)
16. 11 or 12 or 13 or 14 or 15 (1474366)
17. 5 and 10 and 16 (1296)

Database: Cochrane Library databases

1. MeSH descriptor: [Robotic Surgical Procedures] explode all trees (420)
2. robot* surgical procedures:ti,ab,kw (870)
3. MeSH descriptor: [Robotics] explode all trees (1127)
4. robotics:ti,ab,kw (1746)
5. MeSH descriptor: [Surgery, Computer-Assisted] explode all trees (1295)
6. computer assisted surgery:ti,ab,kw (4620)
7. #1 or #2 or #3 or #4 or #5 or #6 (683495)
8. MeSH descriptor: [Thoracic Surgery]
9. erdXCexplode all trees (178)
10. thoracic surgery:ti,ab,kw (10974)
11. MeSH descriptor: [Coronary Artery Bypass] explode all trees (5647)
12. coronary artery bypass:ti,ab,kw (13396)
13. MeSH descriptor: [Heart Valve Prosthesis] explode all trees (567)
14. heart valve prosthesis:ti,ab,kw (1459)
15. ((cardiac or heart) NEAR/2 surg*):ti,ab,kw (14535)
16. #8 or #9 or #10 or #11 or #12 or #13 or #14 (32908)
17. MeSH descriptor: [Learning Curve] explode all trees (204)
18. learning curve:ti,ab,kw (2157)
19. train*:ti,ab,kw (131660)
20. proficien*:ti,ab,kw (2254)
21. competen*:ti,ab,kw (12699)
22. skill*:ti,ab,kw (35443)
23. #16 or #17 or #18 or #19 or #20 or #21 (157118)
24. #7 and #15 and #22 (47)
